# PHYSIOTHERAPEUTIC APPROACHES AND THE EFFECTS ON INSPIRATORY MUSCLE FORCE IN PATIENTS WITH CHRONIC OBSTRUCTIVE PULMONARY DISEASE IN THE PRE-OPERATIVE PREPARATION FOR ABDOMINAL SURGICAL PROCEDURES

**DOI:** 10.1590/0102-672020190001e1439

**Published:** 2019-08-26

**Authors:** Faruk Abrão KALIL-FILHO, Antônio Carlos Ligocki CAMPOS, Elizabeth Milla TAMBARA, Bruna Karoline Alves TOMÉ, Cleiton José TREML, Carlos Henrique KURETZKI, Fernanda Luiza Schumacher FURLAN, Jocilene Pedroso ALBUQUERQUE, Osvaldo MALAFAIA

**Affiliations:** 1 Program of Post-Graduation in Surgical Clinic, Sector of Health Sciences, Federal University of Paraná;; 2 Post-Graduate Program in Principles of Surgery, Evangelic Mackenzie Faculty of Paraná, Curitiba, PR, Brazil

**Keywords:** Chronic obstructive pulmonary disease, Database, Maximum respiratory pressure, Manovacuometry, Doença pulmonar obstrutiva crônica, Banco de dados, Pressão respiratória máxima, Manovacuometria

## Abstract

***Background*:**

Abdominal disorders can alter respiratory function and increase the morbidity and mortality of patients with chronic obstructive pulmonary disease.

***Aim*:**

To improve the physiotherapeutic and muscular capacity in chronic obstructive pulmonary muscular inspiration in the preoperative preparation in abdominal surgeries.

***Method*:**

Retrospective and documentary study using SINPE^*©*^ , clinical database software of patients with chronic obstructive pulmonary disease and candidates to abdominal operation. The sample consisted of 100 men aged 55-70 years, all with chronic obstructive pulmonary disease who underwent preoperative physiotherapeutic treatment. They were divided into two groups of 50 individuals (group A and group B). In group A the patients were treated with modern mobility techniques for bronchial clearance and the strengthening of the respiratory muscles was performed with IMT^*®*^ Threshold. In group B the treatment performed for bronchial obstruction was with classic maneuvers and for the strengthening of the respiratory muscles for flow incentive was used Respiron^*®*^ .

***Results*:**

Both groups obtained improvement in the values ​​of the PiMáx after the different treatments. Group A obtained greater change in the intervals and a more significant increase of the values of the PiMax in relation to the average pre and post-treatment. However, when analyzing the variance and the standard deviation of the samples, group B presented the best results showing more homogeneity.

***Conclusions*:**

The modern and traditional bronchial clearance techniques associated with inspiratory muscle training were equally effective in gaining inspiratory muscle strength with increased Pmax. In this way, the two can be used in the preoperative preparation of patients with chronic obstructive pulmonary disease and referred to abdominal operations.

## RESUMO

***Racional*:**

As operações abdominais podem alterar a função respiratória e aumentar a morbimortalidade dos operados com doença pulmonar obstrutiva crônica.

***Objetivo*:**

Avaliar que técnica fisioterapêutica apresenta melhor efeito na força muscular inspiratória dos pacientes com doença pulmonar obstrutiva crônica no preparo pré-operatório em operações abdominais.

***Método*:**

Estudo retrospectivo e documental usando o SINPE^©^, software de banco de dados clínicos de pacientes portadores de doença pulmonar obstrutiva crônica e candidatos a operações abdominais. A amostra foi composta de 100 homens com idade entre 55-70 anos, todos com DPOC com indicação para operação abdominal e que realizaram tratamento fisioterapêutico pré-operatório. Foram divididos em dois grupos de 50 indivíduos (grupo A e grupo B). No grupo A o tratamento realizado para a desobstrução brônquica foi com técnicas modernas e para fortalecimento dos músculos respiratórios foi realizado o Threshold IMT^®^. No grupo B o tratamento realizado para desobstrução brônquica foi com técnicas tradicionais e para fortalecimento dos músculos respiratórios foi realizado incentivador a fluxo Respiron^®^.

***Resultados*:**

Ambos os grupos obtiveram melhora dos valores da PiMáx (Pressão inspiratória Máxima) após o tratamento com abordagens diferentes com melhora também nos intervalos da PiMáx. O grupo A obteve maior mudança nos intervalos e aumento mais significativo dos valores da PiMáx em relação à média pré e pós tratamento. Porém, ao analisar a variância e o desvio-padrão das amostras, o grupo B apresentou melhor distribuição que o grupo A mostrando que seus resultados na amostra foram mais homogêneos.

***Conclusões*:**

As técnicas modernas e tradicionais de desobstrução brônquica associadas ao treinamento muscular inspiratório mostraram-se igualmente eficazes no ganho de força dos músculos inspiratórios com aumento da PiMáx. Dessa forma as duas podem ser utilizadas no preparo pré-operatório de pacientes com DPOC e encaminhados para operações abdominais.

## INTRODUCTION

The Chronic Obstructive Pulmonary Disease (COPD) is characterized by the limitation of air flow non-totally reversible, progressive and associated to the abnormal inflammatory response in the lungs by inhalation of particles or irritating gases^5^. Many people suffer from this disease for years and die prematurely because of it or its complications. The COPD is the fourth cause of death in the world^21^ and a rise in this placing is expected for the next decades^6^. In 2003, in Brazil, it was the fifth greatest cause of hospitalization in the public system involving people older than 40 and it is nowadays the third cause of death^8^ ^,^ ^10^.

The patients show intolerance to exercise as a consequence of functional alterations of the lungs and of muscular skeletal dysfunction^3^ ^,^ ^13^, rise in the secretion of mucus in air ways and hypertrophy of the mucus producer cells. This set leads to the obstruction of the air flows, increase of resistance to air, shortness of breath, air imprisonment and increase of residual volume, reducing the diaphragm efficiency and reducing the ability to exercise^24^.

Among the factors that can alter the respiratory function in abdominal surgical procedures some stand out: anesthetic drug administration and the anesthesia itself, the manipulation of the entrails, incision in the abdominal cavity, immobilization in bed, muscle relaxants, abdominal distension and pain^22^ ^,^ ^25^. Physiotherapy works in the treatment of acute or chronicle pulmonary disturbances. It has preventive indication of respiratory complications especially in patients submitted to abdominal surgical procedures^18^.

Biomedical Informatics or Health Informatics are defined by Blois and Shorliffe (1990) as a “fast developing scientific field that deals with biomedical information, data and biomedical knowledge to problem solving and decision making”^14^. The software entitled Integrated System of Electronic Protocols (SINPE^©^) serves to the storage and analysis of trustworthy data collected. Its use based in electronics protocols of clinical data allows great storage capacity, crossing and processing of information. It is easy to use and with its automatic back up it optimizes the production of scientific assignments of high quality and reliability.

The objective of this study was to use this computer data storage software to the comparative analysis between physiotherapeutic approaches pointing the ones that had better effect in the inspiratory muscle force while with COPD in the preoperative preparation for abdominal surgical procedures.

## METHODS

This is a retrospective and documental study using SNIPE, a clinical database software of patients with COPD and candidates to abdominal surgery. The sample was composed of 100 men with ages between 55 and 70 years old, all with COPD and with indications to abdominal surgical procedures. All of them performed preoperative physiotherapeutic treatment in service of digestive system surgery in hospital in Curitiba, Paraná, Brazil. They were divided in two groups with 50 individuals (group A and group B), each group performed a certain kind of treatment.

For the bronchial clearance in group A were performed slow expiration with an open glottis (eltgol), acceleration of expiratory flow and forced expiration; for the strengthening of the respiratory muscles the Threshold IMT^®^ has been used.

For the bronchial clearance in group B were performed traditional techniques such as high-frequency chest compression, postural drainage and tapping; for the strengthening of the respiratory muscles the flow encouraging Respiron^®^ has been used.

In both groups, the spirometry was performed before and after 20 interventions in order to evaluate which group would obtain a better effect in the inspiratory muscle force.

 The exclusion criterion were, COPD undiagnosed patients, women, people who were not between 55 and 70 years old and that were not inserted in the SNIPE database.

The data were stored in SINPE in an electronic protocol specifically created for COPD.

### Statistical analysis

For the statistical study the SINPEAnalisador^©^ and free Past softwares were used. The average difference was found through paired T test for group A, with normal distribution and for group B, the Wilcoxon paired test for presenting values under 0.05 in the normality test. Both tests considered a level of significance of 5% p<0.05.

## RESULTS

The ages were considered in five years periods being the youngest 55 and the oldest 70 years old both in groups A and B. In group A, 25 individuals were between 65 and 70 years old. The average age in group A was 63.90 ± 3.97 and in group B, 62.84 ± 4.08.

Figure 2 shows the results obtained in the spirometry with maximum inspiratory pressure in pre- and post-treatment in group A, with values containing 5 cmH_2_O gaps. It can be observed that in pre- treatment, 13 patients were between -50 and -45 cmH_2_O and in post-treatment, only four, showing that nine patients had an improvement in the maximum inspiratory pressure and changed ranges.

**FIGURE 1:**
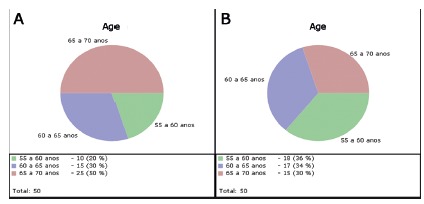
Age gap by patient quantity in groups A and B

**FIGURE 2:**
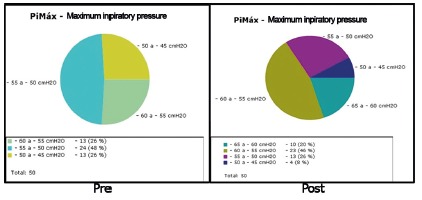
Spirometry of the maximum inspiratory pressure in pre and post treatments in group A.

In the post-treatment anothes gap was inserted, from -65 to -60 cmH_2_O with 10 patients, this gap was nonexistent in pre-treatment because no patients were in this range. That is explained due to the spirometry evaluation that showed an improvement in maximum inspiratory pressure where we can observe that in post-treatment each gap had an alteration in the number of patients.

In the last gap of the pre-treatment group with 13 patients (-60 to -55 cmH_2_O), if we consider the same gap in post-treatment, this number increased to 23 patients, that is, gaps that showed a lower maximum inspiratory pressure and with th intervention they were able to rise in the inspiratoty ranges.

**FIGURE 3:**
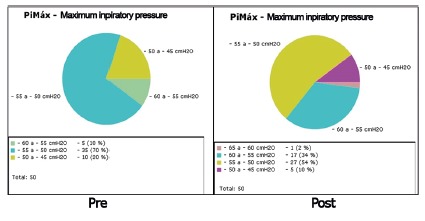
Spirometry of the maximum inspiratory pressure in pre and post treatments in group B

Figure 3 shows the values obtained in the spirometry in maximum inspiratory pressure in pre- and post-treatment in group B with values containing 5 cmH_2_O gaps. It can be observed that in pre-treatment 10 patients were between -50 to -45 cmH_2_O an in post-treatment only five remained in this range and only one was in the -65 to -60 cmH_2_O.

As in Figure 2, it was also inserted the -65 to -60 cmH_2_O in the post-treatment in group B; however, only one patient has able to reach this range, unlike group A that had 10 patients in this range. Likewise, there was an improvement in maximum inspiratory pressure of patients, showing an alteration in the number of patients that came from the lower gaps, that is, gaps that showed a lower maximum inspiratory pressure and with the intervention were able to rise in the inpiratory pressure gaps.

The average of values in group A in the spirometry evaluation of maximum inspiratory pressure in pre-treatment was -52.28 cmH_2_O an in post-treatment -57.5 cmH_2_O, considering the increase in maximum inspiratory pressure after the physiotherapeutic treatment. In group B this evaluation was -51.7 cmH_2_O in pretreatment and -53.6 cmH_2_O, considering also the increasing of values in maximum inspiratory pressure after the physiotherapeutic treatment.

 The standard deviation in the average of values of the maximum inspiratory pressure post treatment of group A was ±3.83 and group B, ±2.86.

It was observed that in group A, as well as in group B, there was an improvement of the values of maximum inspiratory pressure post-treatment with different approaches.

Regarding the change in gap of maximum inspiratory pressure post-treatment it was verified that there was an improvement in both groups. The group that had the biggest change was group A. In groups A and B, 50 patients were in the gaps between -45 and -60 cmH_2_O and none above -60 cmH_2_O. After the treatment and performed a new spirometry of the maximum inspiratory pressure, it was verified that in group A there were 10 patients above -60 cmH_2_O and in group B there was only one.

The group that showed the most meaningful increase regarding the average of the maximum inspiratory pressure values for both pre- and post-treatments was group A submitted to the modern techniques of bronchial clearance and Threshold IMT for the respiratory muscles strengthening. However, analyzing the variation and the standard deviation, group B presented a better distribution of the results than group A, showing that its results in the samples were more uniform.

## DISCUSSION

It was observed, based on the results obtained from the comparative analysis of the maximum inspiratory pressure a meaningful improvement in both groups between the beginning of the treatment and after 20 interventions being then verified a meaningful improvement in the respiratory quality of the participants.

The clearance techniques performed in group A - called modern techniques of bronchial clearance (eltgol, acceleration of the expiratory flow and forced expiratory technique) - are maneuvers that seek to promote the emptying of the existing air in the lungs, facilitating the displacement of the mucus through the expiratory flow. In group B the so called traditional techniques have as an objective the thixotropic effect and the elimination of mucus.

At the present study the techniques helped in the extraction of the mucus since the main symptoms shown by individuals with chronical obstruction are cough, progressive dyspnea and exercise intolerance. This intolerance can be attributed to the limitations in ventilation as well as the skeletal muscle dysfunction developed by chronical deconditioning due to muscle mass reduction being a factor of independent prediction to mortality in these patients^23^.

As for clearance techniques this study could not assert which was the most effective in improving the maximum inspiratory pressure. As for the training of inspiratory muscles with Threshold IMT^®^ and Respiron^®^ for the improvement of maximum inspiratory pressure, both were considered effective in individuals with CPOD, which was pointed by the increase in 30-40% of maximum inspiratory pressure in group A with Threshold as well as in group B with Respiron^®^. The increase in maximum inspiratory pressure was evident in the measurements between pre- and post-intervention in both groups. This fact has also been pointed by Ramirez-Sarmiento et al^17^, that showed that training of the respiratory muscles induces a functional improvement and adaptive changes in these muscles structures.

The patient with obstructive disease presents an increase in airway resistance and aerial imprisonment due to the reduction of the expiratory flow. These two factors determine hyper-inflated lungs, altering the respiratory mechanics with the consequent loss to the respiratory muscles kinetics^20^.

The increase in residual functional capacity caused by the hyper insufflation alters the respiratory mechanics and rectifies the diaphragm. This positioning results in mechanical disadvantage that can be inferred by the reduction in respiratory muscles force.

As for bronchial clearance techniques performed in groups A and B, they do not help pulmonary emptying; however, they presented efficiency in thixotropic effect and mucus elimination.

In studies evaluating the effects of pulmonary emptying in COPD concluded that the pulmonary emptying maneuver provides momentary alterations with the reduction of pulmonary hyper insufflation, however this effect did not keep after exercising^15^.

Many patients with COPD have no reduction of the respiratory muscle force, but due to hyper insufflation they present a reduction of the maximum inspiratory pressure. The main factors that contribute to the respiratory muscle dysfunction in these patients are pulmonary hyper insufflation and the increase in respiratory work force. The low inspiratory pressure gives the prognostic and the severity of the disease^14^.

Abreu *et al*.,^1^ observed that the groups who performed respiratory muscle force training and respiratory muscle endurance presented statistically significant alterations in maximum inspiratory pressure.

As for patients with COPD, two studies showed that the training protocols with loads of 12% and 15% of maximum inspiratory pressure were not effective, though with 30% they were enough to produce the same results as the exercise training^9,11^ resulting in dyspnea reduction, improvement in daily activities performance and reduction of metabolic waste during exercises.

The Threshold IMT^®^ prove itself effective showing an increase in maximum inspiratory pressure and with the rise of this pressure it was possible to see a bigger tolerance to fatigue, which leads to the prevention of possible failures and the improvement of ventilation mechanic and quality of life through the muscular strengthening work force.

In meta-analysis, studies involving patients with COPD show that the inspiratory muscle training significantly increases the strength and the resistance of the inspiratory muscles and diminishes resting dyspnea and during exercise, in accordance with the present study^12^.

The use of Respiron^®^ associated to the bronchial clearance techniques has shown effective in promoting the increasing of force related to the inspiratory musculature and to the increase of maximum inspiratory pressure. This increase for the group that used the Respiron^®^ is explained by the kinesthesia promoted by the technique and still because it induces by the expiration up to the functional residual capacity to maximum inspiration effecting the rise in the spheres with pulmonary deflating^19^.

In studies realized by Freitas and Lima^7^, comparing the use of Threshold IMT^®^ and Respiron^®^ in training ventilation muscles it was concluded that training with both types of respiratory encouragers (linear and not linear) promote the increase in both maximum inspiratory and expiratory pressures.

Despite the different objectives of the Threshold^®^ and Respiron^®^ it is observed in clynical practice that there is an increase in maximum inspiratory pressure (improvement of the respiratory musculature performance) with the resource that is not marketed as a muscular exciter (especific respiratory muscular training).

Azeredo^2^ infers that intense progressive muscular activity starting from the residual volume, increases the alveoli pressure that is directly proportional to the contractile force of the muscles.

The data storage in a database using specific protocol improves the given assistance and the thrustworthiness of the collected data. The electronic protocols favor the data collection in prospective and retrospective forms, increasing the volume of patients gathered data thus improving the scientific knowledge and the volume of published assignments of high scientific value.

The SINPE^©^ performs the search for information directly with the patient and its medical records, gathering data as, for example, of complementary exams, type of surgical or conventional treatment among other information by the multi professional team.

The inferior quality of data gathered through non-computerized medical records may compromise the results in a study, something that does not occur in the collection of data using an electronic database of clinical data because they are stored in a single protocol.

The respiratory physiotherapy electronic protocol for pulmonary disease is an objective quiz, in-depth and of easy filling. It is performed in a structured and elaborated way after extensive review of specific literature. It presents a wide arrange of diseases where physiotherapy acts as well as the physiotherapeutic treatment performed in surgical procedures. It provides in a uniform manner the computerized gathering and storage of clinical data, facilitating the future research of results after the collection of clinical cases for quality and objective scientific researches. This protocol gathers not only basic pulmonary diseases where respiratory physiotherapy acts, but also in pre-operative and post-operative of several surgical procedures.

In face of the realized research, it is suggested that a larger number of interventions was needed for better results, especially concerning the bronchial clearance techniques in COPD patients. Another important factor is in the matter of proof of respiratory function where it would be important to insert spirometry in pre- and post-operative for pulmonary volume evaluation. 

Through this research we expect to contribute with studies accomplished in this area and that other researches use the SNIPE storage protocols, data search and analysis in patients operated with abdominal procedures being able to collaborate with the practical effectiveness of these results.

## CONCLUSION

The modern and traditional techniques of bronchial clearance associated with inspiratory muscle training have shown themselves equally effective in inspiratory muscle force gain with an increase in maximum inspiratory pressure. Thus, both can be used in pre-operative patients with COPD, that are sent to abdominal operations.

## References

[1] Abreu CM, Santos DG, Valle PHC, Costa D (200). Treinamento da musculatura inspiratória em indivíduos normais e
portadores de patologias respiratórias. Fisioter Mov.

[2] Azeredo CAC (2002). Fisioterapia Respiratória Moderna..

[3] Bernard S, Leblanc P, Whitton F, Carrier G, Jobin J, Belleau R (1998). Peripheral muscle weakness in patients with chronic obstructive
pulmonary disease. Am J Respir Crit Care Med.

[4] Blois M., Shortliffe E, Shortliffe E., Perreault L. (1990). The computer meets medicine: emergence of a
discipline. Medical informatics: computer applications in health care.

[5] Consenso Brasileiro sobre Doença Pulmonar Obstrutiva Crônica II -
DPOC (2004). J Bras. Pneumol.

[6] Elliott MW, Adams L, Cockcroft A, MacRae KD, Murphy K, Guz A (1991). The language of breathlessness Use of verbal descriptors by
patients with cardiopulmonary disease. Am Rev Respir Dis.

[7] Freitas V, Lima P (2004). Comparação entre o uso do Respiron e do Threshold no treinamento
dos músculos ventilatórios. Brazilian Journal of Physical Therapy.

[8] Global Initiative for Chronic Obstructive Lung Disease -
GOLD (2011). Global Initiative for Chronic Obstructive Lung Disease.

[9] Gross D., Ladd H. W., Riley E. J., Macklem P. T., Grassino A (1980). The effect of training on strength and endurance of the diaphragm
in quadriplegia. Am J Med.

[10] Jardim J, Oliveira J, Nascimento O (2004). II Consenso Brasileiro de Sobre Doença Pulmonar Obstrutiva
Crônica. J Pneumol,.

[11] Lisboa C., Muñoz V., Beroiza T., Leiva A., Cruz E (1994). Inspiratory muscle training in chronic airflow limitation:
comparison of two different training loads with a threshold
device. EurRespir J.

[12] Lötters F, Van Tol B, Kwakkel G, Gosselink R (2002). Effectsof muscle inspiratory training in patients with COPD a
meta-analysis. Eur Respir J.

[13] Maltais F, Leblanc P, Simard C, Jobin J, Bérubé C, Bruneau J (1996). Skeletal muscle adaptation to endurance training in patients with
chronic obstructive pulmonary disease. Am J Respir Crit Care Med.

[14] Mckenzie DK, Butler JE, Gandevia SC (2009). Respiratory muscle function and activation in chronic obstructive
pulmonary disease. J Appl Physiol.

[15] Payno S. M., Clemente S. K., Capretz S. M., Terra João (2007). Efeitos da desinsuflação pulmonar nos portadores de doença
pulmonar obstrutiva crônica (dpoc). Rev. Bras. Fisioter.

[16] Pereira EDB., Farensin SM., Fernandes ALG (2000). Morbidade respiratória nos pacientes com e sem síndrome pulmonar
obstrutiva submetidos a cirurgia abdominal alta. Rev. Assoc. Med.Bras.

[17] Ramirez-Sarmiento A, Orozco-Levi M, Güell R, Barreiro E, Hernandez N, Mota S (2002). Inspiratory muscle training in patients with chronic obstructive
pulmonary disease structural adaptation and physiologic
outcomes. Am J Respir Crit Care Med.

[18] Rocha MRS, Souza S, Costa CM, Merino DFB, Montebelo MIL, Rasera-Júnior I (2018). Airway positive pressure vs. exercises with inspiratory loading
focused on pulmonary and respiratory muscular functions in the postoperative
period of bariatric surgery. ABCD, arq. bras. cir. dig.

[19] Sarmento GJV (2007). Fisioterapia respiratória no paciente crítico: rotinas clínicas.

[20] Sharp JT (1986). The respiratory muscles in chronic obstructive pulmonary
disease. Am Rev Respir Dis.

[21] Simon PM, Schwartzstein RM, Weiss JW, Fencl V, Teghtsoonian M, Weinberger SE (1990). Distinguishable types of dyspnea in patients with shortness of
breath. Am Rev Respir Dis.

[22] Simonneau G, Vivien A, Sartene R (1983). Diaphragm dysfunction induced by upper abdominal surgery Role of
postoperative pain. Am Rev Respir Dis.

[23] Tarantino AB (1997). Doenças pulmonares.

[24] Valderramas SR, Atallah AN (2009). Effectiveness and safety of hypertonic saline inhalation combined
with exercise training in patients with chronic obstructive pulmonary
disease a randomized trial. Respir Care.

[25] Westbrook PR, Stubbs SE, Sessler AD (1973). Effects of anesthesia and muscle paralysis on respiratory
mechanics in normal man. J Appl Physiol.

